# Classic and new strategies for the treatment of advanced melanoma and non-melanoma skin cancer

**DOI:** 10.3389/fmed.2022.959289

**Published:** 2023-02-09

**Authors:** Marco Rubatto, Nadia Sciamarrelli, Silvia Borriello, Valentina Pala, Luca Mastorino, Luca Tonella, Simone Ribero, Pietro Quaglino

**Affiliations:** Department of Medical Sciences, Dermatologic Clinic, University of Turin, Torino, Italy

**Keywords:** melanoma, non-melanoma skin cancer, immunotherapy, new drugs, targeted therapy

## Abstract

Advanced melanoma and non-melanoma skin cancers (NMSCs) are burdened with a dismal prognosis. To improve the survival of these patients, studies on immunotherapy and target therapies in melanoma and NMSCs are rapidly increasing. BRAF and MEK inhibitors improve clinical outcomes, and anti-PD1 therapy demonstrates better results than chemotherapy or anti-CTLA4 therapy in terms of the survival of patients with advanced melanoma. In recent years, the combination therapy of nivolumab plus ipilimumab has gained ground in studies for its survival and response rate benefits in patients with advanced melanoma. In addition, neoadjuvant treatment for stages III and IV melanoma, either as monotherapy or combination therapy, has recently been discussed. Another promising strategy evaluated in recent studies is the triple combination of anti-PD-1/PD-L1 immunotherapy and anti-BRAF plus anti-MEK targeted therapy. On the contrary, in advanced and metastatic BCC, successful therapeutic strategies, such as vismodegib and sonidegib, are based on the inhibition of aberrant activation of the Hedgehog signaling pathway. In these patients, anti-PD-1 therapy with cemiplimab should be reserved as the second-line therapy in case of disease progression or poor response. In patients with locally advanced or metastatic SCC, who are not candidates for surgery or radiotherapy, anti-PD1 agents such as cemiplimab, pembrolizumab, and cosibelimab (CK-301) have shown significant results in terms of response rate. PD-1/PD-L1 inhibitors, such as avelumab, have also been used in Merkel carcinoma, achieving responses in half of the patients with advanced disease. The latest prospect emerging for MCC is the locoregional approach involving the injection of drugs that can stimulate the immune system. Two of the most promising molecules used in combination with immunotherapy are cavrotolimod (a Toll-like receptor 9 agonist) and a Toll-like receptor 7/8 agonist. Another area of study is cellular immunotherapy with natural killer cells stimulated with an IL-15 analog or CD4/CD8 cells stimulated with tumor neoantigens. Neoadjuvant treatment with cemiplimab in CSCCs and nivolumab in MCCs has shown promising results. Despite the successes of these new drugs, the new challenges ahead will be to select patients who will benefit from these treatments based on biomarkers and parameters of the tumor microenvironment.

## Introduction

Melanoma is a severe form of skin cancer, and non-melanoma skin cancers (NMSCs) comprise a heterogeneous group of cancers.

Major NMSCs include basal cell carcinoma (BCC), cutaneous squamous cell carcinoma (cSCC), and Merkel cell carcinoma (MCC). The most frequent are cSCC and BCC, while MCC has a lower incidence (1). SCC and BCC have better prognoses, unlike MCC.

NMSCs arise in elderly patients, while melanoma is one of the most common cancers in young people; however, its incidence increases with age.

In terms of incidence, in the United States, it is the fifth most frequent cancer in both genders (2).

Early diagnosis has a crucial role in the survival rates, as a patient's prognosis depends on the stage of the disease at the time of the diagnosis. Nowadays, most patients present with localized disease, which can be treated with surgical excision.

However, as these cancers become metastatic, the prognosis is poor in melanoma and in all three types of NMSC. Metastatic melanoma had a 3-year overall survival (OS) rate that was relatively constant from 2004 to 2009, ranging from 26.4% to as low as 4.7% across the subcategories of stage IV metastatic disease (3).

The median OS period of metastatic BCC has been reported to be 10.0 months (range 0.5–108.0 months) after metastases detection (4). The median OS of metastatic cSCC patients has been reported to be 2.19 years, and the 5-year survival rate was 26% in USA (5). Finally, patients with metastatic MCC (mMCC) have the worst prognosis, with a historical 5-year overall survival (OS) rate of ≤18% (6). To overcome this major pitfall, promising new drugs are being used to improve the prognosis of these patients.

Studies regarding immunotherapy in melanoma and NMSCs are recent and rapidly increasing. Melanoma and NMSCs are characterized by significant expression of the PD-1/PD-L1 axis in both tumor tissues and infiltrating immune cells. Thus, patients may benefit from immunotherapy treatment (7).

Beyond immunotherapy, targeted therapies have also revolutionized the therapeutic approach to advanced skin diseases.

BRAF is mutated in around 50% of melanomas. The therapeutic landscape for this tumor has broadened with the development of BRAF inhibitor (8).

**Figure 1:**
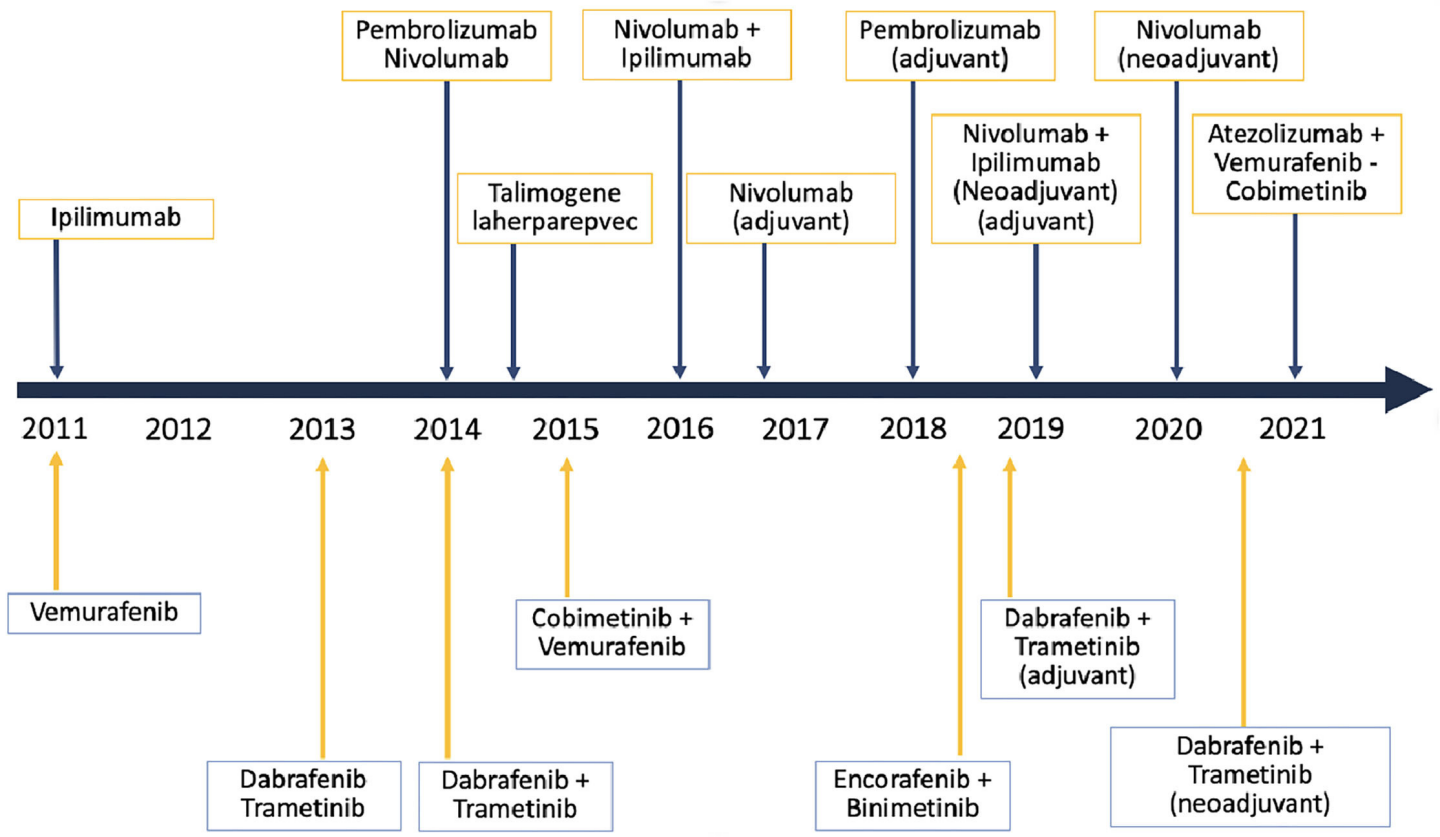
Timeline of target therapies and immunotherapy in melanoma.

An aberrant activation of the Hedgehog signaling pathway is implicated in the pathogenesis of BCC. Notably, Hedgehog signaling inhibitors, such as vismodegib and sonidegib, are successfully used as a targeted treatment for advanced or metastatic BCC (9) (Figure 1).

## BRAF and MEK inhibitors

The most common genetic mutation in melanoma is the V600E/K mutation of the BRAF gene, which appears in almost 50% of cutaneous and 10–20% of mucosal melanomas (10).

These alterations promote cellular survival and replication in tumors and are caused by the constitutional activation of BRAF kinase, which is crucial in mitogen-activated protein kinase (MAPK) signaling, including MEK1 and MEK2 kinase.

BRAF and MEK inhibitors are small molecules that cause inhibition of these proteins, and for this effect, they have been approved as a first-line therapy for melanoma since 2010 (11).

These molecules, among others, have contributed in the last years to changing the OS prognosis, especially in unresectable stages (stages III/IV) that have < 12 months OS prognosis without therapy (12).

The combination targeted therapy with BRAF/MEK inhibitors that have received Food and Drug Administration (FDA) approval as first-line treatment in the unresectable setting are dabrafenib plus trametinib (DAB+TRAM), vemurafenib plus cobimetinib (VEM+COBI), and encorafenib plus binimetinib (ENCO+BINI) (13).

Dabrafenib plus trametinib is also recommended as combined adjuvant therapy for 52 weeks after resection in IIIA/B/C/D BRAF-mutant (V600E/K^*^) melanomas (14).

During the past years, several studies among patients with BRAF V600 E/K mutations were performed.

The COMBI-AD trial included patients with resected stages IIIA-IIIC melanoma treated with dabrafenib plus trametinib and had a placebo in the comparator arm. Results showed significant improvement in RFS (HR, 0.47; 95% CI, 0.39–0.58; *P* = 0.001) and OS (HR, 0.57; 95% CI, 0.42–0.79; *P* = 0.0006) after a minimum follow-up of 3 years (15).

Furthermore, three randomized phase III trials comparing combination targeted therapy vs. BRAF inhibitor monotherapy were conducted among patients with unresectable BRAF V600 mutated melanoma: COMBI-v (DAB+TRAM vs. vemurafenib) (16), coBRIM (VEM+COBI vs. vemurafenib) (17), and COLUMBUS (ENCO+BINI vs. encorafenib and vemurafenib) (18).

According to the results, combination therapy with BRAF and MEK inhibitors is confirmed to improve clinical outcomes. More specifically, PFS was similar for each combination, with better results observed for encorafenib/binimetinib (PFS: ENCO+BINI, 14.8 months; VEM+COBI, 12.3 months; DAB+TRAM, 11.4 months; median OS: ENCO+BINI, 33.6 months; VEM+COBI, 22.3 months; and DAB+TRAM, 25.6 months) (19).

Moreover, the COLUMBUS trial demonstrated the superiority of encorafenib monotherapy vs. vemurafenib monotherapy in OS and PFS.

In addition, Zengarini et al. evaluated the different responses to systemic therapies and the different prognoses between melanomas with BRAF V600E and BRAF V600K mutations.

This study found that BRAF V600K mutated melanomas had a lower response rate to therapies and a greater and more rapid tendency to metastasize, with greater resistance to therapy resulting in a higher and more rapid mortality rate during follow-up (20).

## Anti-CTLA4

Cytotoxic T lymphocyte-associated protein 4 or CTLA-4 (CD152) is a receptor expressed by CD4+ and CD8+ T cells. CTLA-4 binding with CD80 (B7-1) and CD86 (B7-2) ligands on the antigen-presenting cells (APCs) leads to suppression of T-cell responses for causes that are still not fully explained. In Treg cells, this receptor results in constitutively activated, as they represent an immune checkpoint that regulates T-cell homeostasis and self-tolerance (21).

Since its approval by the FDA in 2011 for the treatment of advanced melanoma, CTLA-4 inhibitor ipilimumab has gained space in other solid tumors therapy. Indeed, CTLA-4 block by an antibody releases effector T cells from inhibition, resulting in their activation against cancer cells (22).

Ipilimumab registration derives from a study in which ipilimumab associated with dacarbazine was compared to dacarbazine plus placebo in patients with metastatic melanoma without previous therapy, with significantly better results in the first group in terms of OS (11.2 vs. 9.1 months), survival rates at 1 year (47.3 vs. 36.3%), 2 years (28.5 vs. 17.9%), and 3 years (20.8 vs. 12.2%) (hazard ratio for death, 0.72; *P* < 0.001) (23).

According to the latest ASCO guidelines, ipilimumab in association with nivolumab, followed by nivolumab monotherapy, is recommended for unresectable/metastatic cutaneous melanoma, alternatively to anti-PD1 or BRAF inhibitors and MEK inhibitors (the latter in the case of BRAF V600 mutated melanoma). Ipilimumab is also recommended for unresectable/metastatic melanoma that has progressed after anti-PD1 therapy. In the case of BRAF V600 mutated melanoma, it can be offered instead of BRAF/MEK inhibitor combined therapy or after tumor progression on the latter therapy (14).

These findings are confirmed by the systematic review conducted recently by Alrabadi et al. which included 15 studies, of which 10 included patients in therapy with ipilimumab and who had previously progressed on anti-PD-1. This meta-analysis concludes that a response to a different type of immunotherapy as ipilimumab is possible in anti-PD-1 refractory patients, indeed. On the contrary, results in terms of ORR were in general inferior compared to the same therapy in anti-PD-1-naïve patients, with the most favorable responses obtained with combined nivolumab plus ipilimumab therapy (ORR 23.08%, 95% CI: 16.75–30.03%), followed by ipilimumab monotherapy (ORR 8.19%, 95% CI: 5.78–10.92%). Also, the medium OS in these studies resulted in inferiority with ipilimumab (mOS: 5.1–7.4 months) in comparison with ipilimumab in anti-PD-1 naive patients (24).

## Anti-PD-1

Programmed cell death protein 1 (PD-1) is expressed on the membrane of lymphocytes, where it binds the programmed cell death ligand 1 (PD-L1 or B7-H1). This interaction leads to the inhibition of cell apoptosis and the transition of effector T cells to Treg cells (25).

PD-1 expression on tumor-infiltrating lymphocytes and the presence of its ligands on tumor and immune cells in the tumor microenvironment counteract the antitumoral response, supporting the use of PD-1 inhibition in cancer treatment (26).

Antibodies anti-PD-1, such as pembrolizumab and nivolumab, together with anti-CTLA-4, are indeed part of the group of antineoplastic drugs defined as immune checkpoint inhibitors (ICIs). Anti-PD1 agents have been approved by the FDA in the last decade for a number of clinical indications, including advanced melanoma (27).

According to the recent ASCO guidelines, the first choice as adjuvant therapy for patients with resected stage IIIA/B/C BRAF wild-type melanoma consists of 52 weeks of nivolumab or pembrolizumab. For patients with unresectable/metastatic cutaneous melanoma, the first-line therapy is nivolumab, either in combination with ipilimumab or alone, or, alternatively, pembrolizumab.

In patients with BRAF V600 E/K mutated melanoma, anti-PD1 is used as an alternative to the BRAFi+MEKi combination in both the adjuvant and therapeutic settings (14).

These guidelines are derived from results obtained through several trials conducted in recent years. First, anti-PD1 monoclonal antibodies against chemotherapy have been evaluated in many studies.

CheckMate 066 was a phase III trial, which compared nivolumab monotherapy vs. dacarbazine as the first-line treatment for BRAF wild-type naive for the treatment of advanced melanoma. It demonstrated significantly superior OS in patients receiving nivolumab (72.9 vs. 42.1%, respectively) (28).

CheckMate 037 was a phase III trial of nivolumab vs. investigators' choice chemotherapy (ICC) in ipilimumab-refractory patients with advanced melanoma. First, the percentage of patients who had a confirmed objective response was found to be significantly increased in the nivolumab arm (31.7%, 95% CI: 23.5–40.8 vs. 10.6%, 3.5–23.1, respectively) (29). Subsequently, median overall survival was also assessed in favor of the nivolumab-treated group (16 vs. 14 months, respectively; HR, 0.95; 95.54% CI: 0.73–1.24) (30).

Keynote-002 was a phase II trial comparing pembrolizumab 2 mg/kg vs. pembrolizumab 10 mg/kg (both every 3 weeks) vs. ICC in ipilimumab-refractory patients. PFS was incremented in the pembrolizumab 2 mg/kg arm (HR 0.57, 95% CI: 0.45–0.73; *p* < 0.0001) and in the pembrolizumab 10 mg/kg group (0.50, 0.39–0.64; *p* < 0.0001), compared with the ICC arm. A 6-month progression-free survival was 34% (95% CI: 27–41) in the first group, 38% (31–45) in the second group, and 16% (10–22) in the latter group (31).

In addition, anti-PD1 therapy has been compared with anti-CTLA4 therapy in several studies.

Keynote-006 study of pembrolizumab vs. ipilimumab in patients with advanced melanoma (32) showed superior median overall survival in the pembrolizumab-treated group (32.7 months; 95% CI: 24.5–41.6) and 15.9 months (13.3-22-0) in the ipilimumab group (HR 0.73, 95% CI 0.61–0.88, *p* = 0.00049) (33).

Checkmate 067 was a phase III study that included two groups of previously untreated patients who received nivolumab or ipilimumab as monotherapy, respectively, and also an arm receiving nivolumab plus ipilimumab (N+I) as combination therapy. It demonstrated a median PFS increase of 11.5 months (95% CI: 8.7–19.3) in the combination therapy arm vs. a PFS of 6.9 months (5.1–10.2) in the nivolumab arm and 2.9 months (2.8–3.2) in the ipilimumab arm (34). A 5-year overall survival was greater in the combination therapy arm than in the nivolumab and ipilimumab groups (52 vs. 44 vs. 26%, respectively) (35).

A meta-analysis of 16 studies including N+I therapy and nivolumab monotherapy was recently conducted by Xu Y et al.

According to the data found, combination therapy provided benefits in terms of ORR [RR = 1.40 (95% CI: 1.27, 1.54), *P* < 0.00001] and PFS [HR = 0.83 (95% CI: 0.77, 0.90), *P* < 0.00001].

In addition, different doses of the N+1 combination were evaluated: nivolumab 1 mg/kg plus ipilimumab 3 mg/kg (N1I3) and nivolumab 3 mg/kg plus ipilimumab 1 mg/kg (N3I1).

The N1I3 combination was more effective in terms of ORR, PFS, and OS but was associated with a higher incidence of high-grade AEs (36).

Also, the current clinical practice of choosing on an individual basis between pembrolizumab and nivolumab in advanced melanoma is confirmed by a retrospective study conducted by Moser JC et al. Indeed, in that study, the median OS for patients treated with first-line pembrolizumab was 22.6 m (IQR 6.8-NR), whereas the median OS for those treated with nivolumab was 23.9 m (IQR 6.8–39.5, *p* = 0.91), demonstrating comparable efficacy of pembrolizumab and nivolumab in this setting (37).

Furthermore, recent studies have investigated pembrolizumab therapy in an adjutant setting:

Phase III EORTC 1325-MG/KEYNOTE-054 study of pembrolizumab vs. placebo in patients with resected high-risk stage III cutaneous melanoma demonstrated that RFS was longer in the pembrolizumab group (59.8%, 95% CI: 55-3-64-1) than in the placebo group (41.4%, 37.0–45.8) at 3.5 years of follow-up (HR 0–59, 95% CI: 0.49–0.70). It also settled that the RFS in the PD-L1-positive pembrolizumab subgroup was greater (61.4%, 56.3–66.1) than in the PD-L1-positive placebo subgroup (44.1%, 39.2–48.8; HR 0–59, 95% CI: 0.49–0.73) (38).

KEYNOTE-716 phase III trial of pembrolizumab vs. placebo as adjuvant therapy in patients with completely resected, high-risk, stage II melanoma. At the first intermediate analysis, it was determined that recurrence or death was reduced in patients in the pembrolizumab group: 11 vs. 17% (hazard ratio [HR] 0.65, 95% CI: 0.46–0.92; *p* = 0.0066). This finding was also confirmed in the second interim analysis in which the occurrence of this event was 15% in the first group and 24% in the second one (HR 0.61, 95% CI: 0.45–0.82) (39).

## LAG-3-targeted therapies

Lymphocyte-Activation Gene 3 (LAG-3) is one of the most important next-generation immune checkpoint molecules (40–42), having a similar function to PD-1 and CTLA-4 (43, 44).

Having a proapoptotic role on T cells, this molecule is considered to be a marker of aggressiveness, poor prognosis, and reduced survival in many cancers. Moreover, its expression correlates with mechanisms of resistance to anti-PD-1/anti-PD-L1 immunotherapy (45–47).

For this reason, anti-LAG-3 inhibitors are a considerable subject of study both alone and in combination with anti-PD1 inhibitors.

As a matter of fact, 108 phase I, I/II, II, II/III, and III studies are underway evaluating different types of LAG-3-targeted molecules alone or in combination with anti-PD1 inhibitors (48).

In addition, in February of this year, the drug Opdualag, a combination of the anti-LAG-3 antibody Relatlimab with nivolumab, received FDA approval for unresectable or metastatic melanoma (ref. FDA). This milestone was made possible by data from the phase II-III, global, double-blind, randomized RELATIVITY-047 study.

This milestone was made possible by data from the phase II-III, global, double-blind, randomized RELATIVITY-047 study, which compared the combination of relatlimab plus nivolumab as a fixed dose with nivolumab alone. The study showed that this combination, compared with nivolumab monotherapy, more than doubles the PFS of patients with previously untreated metastatic or unresectable melanoma. In fact, the median progression-free survival was 10.1 months (95% CI, 6.4–15.7) with relatlimab-nivolumab compared with 4.6 months (95% CI, 3.4–5.6) with nivolumab (hazard ratio for progression or death, 0.75 [95% CI, 0.62–0.92]; *P* = 0.006 by log-rank test) (49).

## New perspectives on melanoma

### Neoadjuvant setting

One of the possible therapeutic strategies that have been discussed recently is the eventual use of neoadjuvant systemic therapy in certain subgroups of patients with advanced melanoma. Currently, outside of clinical trials, neoadjuvant systemic therapy is not routinely recommended by guidelines for patients with regional metastatic or distantly resectable cutaneous melanoma (14).

Theoretically, neoadjuvant therapy could lead to several advantages. The possibility to make inoperable tumors operable with curative intent and/or to increase the rate of R0 resections (50).

Moreover, it could allow the efficacy of immunotherapy to be established in the patient for possible subsequent adjuvant treatment. Also, T cell checkpoint blockade could be more effective in a neoadjuvant than in an adjuvant setting due to the presence of a larger tumor mass that could increase the T cell response (51).

On the contrary, a disadvantage of this setting could be the delay of the standard-of-care surgery that could render initially operable patients inoperable due to disease progression (52).

This topic was discussed in the meta-analysis published by Erstad et al. in the past months, which concludes that preliminary trial data are currently supportive of the use of neoadjuvant therapy for stage III and IV melanoma (53).

In particular, the results of multiple trials were analyzed: regarding BRAF V660 mutated melanomas, the clinical trial number NCT02231775 of the standard of care group (surgery and subsequent adjuvant therapy) vs. neoadjuvant plus adjuvant dabrafenib and trametinib were conducted. It demonstrated that median event-free survival was 19.7 months in the neoadjuvant arm vs. only 2.9 months (HR 0.016, 95% CI 0.00012–0.14, *p* < 0.0001) in the standard of care group (54).

These results were in line with those obtained from the phase II NeoCombi trial in patients with BRAF-mutated stage IIIB or IIIC melanoma treated with dabrafenib plus trametinib and subsequent surgical resection. A complete pathological response was registered in 49% of patients (5% CI: 31–66) and in 51% of patients (95% CI: 34–69), a non-complete pathological response was observed (55).

Furthermore, Amaria et al. investigated neoadjuvant PD-1 monotherapy with nivolumab vs. combination therapy with nivolumab plus ipilimumab in patients with resectable high-risk melanoma. This trial highlighted that combined therapy shows higher ORR (73%) and pCR (45%) for the price of higher toxicity (73% grade 3 trAEs). On the contrary, nivolumab monotherapy shows reduced ORR and pCR (both results of 25%), but with only 8% grade 3 trAEs (52).

Regarding data comparing therapy in adjuvant and neoadjuvant settings, the OpACIN study was analyzed. It compared combination therapy with ipilimumab plus nivolumab in the adjuvant vs. neoadjuvant setting. The 4-year EFS rate was higher in patients treated with perioperative therapy than in those treated with adjuvant therapy (80 v. 60%, respectively). Furthermore, the 4-year OS rate was 90% in the first group compared with only 70% in the latter group (51).

There are currently five ongoing phase I and II trials evaluating the best combination of immunotherapy in neoadjuvant settings for melanoma (Table 1).

**Table 1:** Ongoing neoadjuvant trials with checkpoint inhibition in melanoma.

**Trial**	**Design**	**N' of patients**	**Intervention**	**Pathologic complete response**
**Trials comparing adjuvant and neoadjuvant checkpoint inhibition**				
***NCT02437279*** ***(OpACIN)***	Phase Ib	20	Arm A: Adjuvant ipi + nivo for 4 cycles	Arm A: N/A
			Arm B: Neoadjuvant ipi + nivo for 2 Cycles before surgery, and 2 after surgery	Arm B: 30%
**Trials with only neoandjuvant arms**				
***NCT02519322***	Phase II	23	Arm A: Neoadjuvant nivo up to 4 cycles, adjuvant nivo up to 13 cycles	Arm A: 25%
			Arm B: Neoadjuvant ipi + nivo up to 3 cycles, adjuvant nivo up to 13 cycles	Arm B: 45%
***NCT02434354***	Phase I	29	200 mg of pembrolizumab, single cycle 3 weeks prior to	18,50%
			surgery then pembrolizumab q3w for a year following surgery	
***NCT02977052 (OpACIN-neo)***	Phase II	86	Arm A: Neoadjuvant ipi (3 mg/kg) + nivo (1 mg/kg) for 2 cycles	Arm A: 47%
			Arm B: Neoadjuvant ipi (1 mg/kg) + nivo (3 mg/kg) for 2 cycles	Arm B: 57%
			Arm C: Neoadjuvant ipi (3 mg/kg) for 2 cycles followed by neoadjuvant nivo (3 mg/kg) for 2 cycles	Arm C: 23%
***NCT02977052***	Phase II	99	Neoadjuvant ipi + nivo for 6 weeks, target node resection, if pCR, no lymphadenectomy,	61% MPR
			if pPR, lymphadenectomy only, if no response, lymphadenectomy + adjuvant nivo for 52 weeks	
**Ipi, ipilimumab; MPR, major pathologic response, defined as < 10% viable tumor cells; nivo, nivolumab; pCR, pathologic complete response**.				
***NCT02231775***	Phase II	21	Arm A: Upfront surgery with consideration of adjuvant therapy	Arm A: NA
			Arm B: Neoadjuvant dabrafenib and trametinib for 8 weeks followed by adjuvant dabrafenib and trametinib for 44 weeks	Arm B: 50%
***NCT01972347 (Neo Combi)***	Phase II	35	Neoadjuvant dabrafenib and trametinib for 12 weeks, adjuvant therapy for 40 weeks	49%
**Trials with only neoadjuvant arms**				
***NTR4654***	Phase II	20	Neoadjuvant dabrafenib and trametinib for 8 weeks in patients with unresectable disease followed by surgery if resectable	28.6%

### Triple combination therapy

Another promising strategy evaluated in recent trials is the triple combination of anti-PD-1/PD-L1 immunotherapy and BRAF inhibitors plus MEK inhibitors targeted.

The rationale for this proposal stems from the fact that immunotherapy and target therapy are complementary, with the latter increasing the upregulation of PD-1/PD-L140. Therefore, the addition of anti-PD-1/PD-L1 to BRAF and MEK targeted therapy could help prevent tumor progression.

This therapeutic alternative was evaluated by Liu Y et al. in patients with stage III-IV melanoma, in a meta-analysis of five randomized controlled trials, for a total of 1,266 patients treated with triple therapy vs. a combination of two drugs or monotherapy as a control group.

A higher OS rate was found in the triple therapy arm compared with the control arm (63.7 vs. 56.3%, respectively), as was the overall ORR (67.1 vs. 62.7%, respectively).

However, triple combination therapy was aggravated by a higher incidence of risk for hypothyroidism, arthralgias, myalgias, increased ALT and AST, asthenia, and fever compared with the control group.

For these reasons, this meta-analysis concludes that currently further controlled trials are required to indicate eventual three combination therapy (56).

Currently, trials are available for evaluating the most favorable sequence to assess toxicity, and some phase II and III trials are for evaluating concomitant triple therapy.

## BCC

Only in a small percentage of patients, BCC can progress to an advanced stage and even more rarely to a metastatic stage (mBCC).

Vismodegib and sonidegib, inhibitors of the hedgehog pathway, have been approved for the treatment of advanced BCC. According to Gutzmer's (57), study vismodegib has a response in 40–70% of metastatic or locally advanced BCC cases. The usual dose is 150 mg PO (until disease progression or toxicity is acceptable). According to this study, 69% of the responses were maintained at a median follow-up of 18 months, with complete response rates of 33% and partial response rates of 35% (57). The median progression-free survival has been reported to be 23 months. In this study, 1/3 of patients discontinued therapy due to side effects. Major side effects included myalgias, dysgeusia, weight loss, and alopecia (56). Moreover, sonidegib had a similar response rate to vismodegib. The usual dose is 200 mg PO (until disease progression or toxicity appears). The main side effects appeared to be muscle spasms with myalgias, alopecia, dysgeusia, and weight loss (57). Vismodegib, unlike sonidegib, is FDA-approved for both locally advanced BCC and metastatic BCC and is not suitable for locoregional therapy. Sonidegib is not approved for metastatic BCC but only for inoperable locally advanced BCC or BCC relapses treated with surgery and RT (58). On the contrary, vismodegib is also used in Gorling Goltz syndrome.

Currently, no direct comparison trials between the two Hedgehog pathway inhibitors have been conducted (59). However, according to data on different response rates, vismodegib would appear to have greater efficacy than sonidegib in mBCC, and therefore, it presents this indication (60).

Because BCCs also express PD-L1, Cemiplimab has been shown to prevent tumor cells from using the PD-1/PD-L1 binding-mediated signaling pathway to suppress T-cell activation. The use of immunotherapy seems to be useful in syndromic forms such as Gorlin-Goltz syndrome. It is possible to initiate therapy with PD-1 inhibitors when it is impossible to initiate therapy with Hedgehog inhibitors or for possible intolerances (61).

For patients with locally advanced basal cell carcinoma after hedgehog inhibitor therapy, Cemiplimab demonstrated clinically significant antitumor activity, according to results from an open-label, single-arm phase II trial (NCT03132636) that were published in *Lancet Oncology*. The primary end point of objective response assessed by independent central review (ICR) was observed in 31% (95% CI, 21%-42%) of patients in the study population. In total, 6% of patients experienced a complete response, and 25% experienced a partial response.

In addition, anti-PD-1 therapy is to be reserved in case of disease progression or in case of poor response to 9-month therapy with Hedgehog pathway inhibitors.

In the study by Stratigos et al., cemiplimab therapy achieved 31% response in patients with locally advanced BCC, including 6% complete and 25% partial responses, and 79% of patients maintained a response at 6 months or longer (62).

Table 2 lists the main ongoing trials of BCC and experimental therapies.

**Table 2:** Ongoing trials in BCC.

**Trial**	**Design**	**N' of patients**	**Indication**	**Intervention**	**ORR**
***NCT02690948***	Phase I/II	16	Advanced BCC	Participants who previously received vismodegib and subsequently progressed will receive pembrolizumab IV over 30 min on day 1. Cycles are every 21 days for up to 24 months in the absence of disease progression or unacceptable toxicity.	ORR 44%
				Participants who have not progressed while receiving vismodegib will receive pembrolizumab IV over 30 min on day 1 and take vismodegib 150 mg by mouth daily. Cycles repeat every 21 days for up to 24 months in the absence of disease progression or unacceptable toxicity.	ORR 29%
***NCT04679480***	Phase II	20	Advanced BCC	Cemiplimab administered as a flat 350 mg dose in every 3 weeks. Sonidegib 200 mg capsule, orally administered once daily. Sonidegib will be administered in a 2 week cycle every 4 weeks starting from week 0.	
***NCT03521830***	Phase II	40	laBCC/mBCC	Arm A: Nivolumab 480 mg IV every 4 weeks	
				Arm B: Ipilimumab 1 mg/kg IV every 4 weeks for 4 doses	
				Arm C: Relatlimab 480 mg IV q4wks	
***NCT04323202***			Advanced BCC of head/neck		
***NCT04799054***			Locally advanced/metastatic solid tumors		
***NCT03458117 (20139157 T-VEC)***			Locally advanced NMSC		
***NCT02978625***			Advanced/refractory NMSC		

## SCC

Cutaneous squamous cell carcinoma is the second most frequent skin cancer in NMSCs (1). The main treatment is surgical excision with histopathologic control of the resection margins, with a cure rate reaching up to 95%. Radiation therapy is the second treatment of choice if surgery is not indicated. In advanced or metastatic SCC, chemotherapeutic drugs such as cisplatin and EGF receptor inhibitors such as cetuximab have been used in the past, unfortunately with a short duration of response and poor survival (63). Advanced spinocellular carcinoma is defined as a stage III, IVa, or IVb tumor according to the TNM classification, or in general, an SCC in which surgery or radiotherapy is not indicated. According to this definition, even a stage I-II or III, if inoperable, is considered “locally advanced.”

For patients with locally advanced or metastatic SCC who are not candidates for surgery or radiation therapy, anti-PD1 drugs have shown relevant results (64). One of the first drugs used was cemiplimab, which was approved by the FDA in September 2018 (65) and in June 2019 by the EMA for mcSCC and lacSCC (66). The approved dose is 350 mg intravenously over 30 min every 3 weeks. Cemiplimab is a highly effective, entirely human monoclonal antibody IgG4 against the PD-1 receptor (67).

Cemiplimab can enhance T-cell responses, including anti-tumor responses, by blocking the binding of PD-1 to the ligands PD-L1 and PD-L2. Cemiplimab is estimated to give ~46.1% response in metastatic patients with SCC, of which 16.1% is considered to be a complete response. The median response time is 2.1 months (NCT02760498).

At 24 months, responsive patients are 69.4%, with disease-free survival at 18.4 months. At 2 years, an estimated 73.3% of patients treated with cemiplimab are alive. Patients who have never undergone surgery respond better than those who have undergone surgery several times (50 vs. 24%) (68). The main adverse events are similar to those of the other immunotherapy drugs, and only 7% of patients show side effects that require discontinuation of treatment. The main adverse effects appear to be fatigue, diarrhea, itching, nausea, and cough (69).

Given the rapidity of cemiplimab to achieve response and its long-term maintenance, it would seem possible to use a short-term therapy scheme for the treatment of patients with lacSCC. Data have been presented in this regard by Conforti C et al., who report their experience of treatment with 3 cycles of cemiplimab 350 mg in two patients who maintained a clinical dermoscopic response at 6 months after discontinuation of treatment (70).

Other immunotherapy drugs are also being studied for the treatment of advanced or metastatic SCC. Keynote 629 (NCT02964559) is a phase II study in which pembrolizumab was administered at 200 mg every 3 weeks in patients with unresectable or metastatic cSCC. Pembrolizumab obtained an objective response rate (ORR) of 50% (*n* = 54; 95% CI: 36.64): a complete response rate of 17% and a partial response rate of 33%. Moreover, among those who responded (*n* = 27), 81% experienced a duration of response (DOR) lasting 6 months or longer, and 37% experienced a DOR lasting 12 months or longer (71). In addition, a French study used pembrolizumab in the same program (NCT02883556) in unresectable cSCC without prior systemic therapy and was also reported at the 2019 ASCO meeting. Considering a total of 39 patients, 2 patients achieved a complete response and 13 patients achieved partial responses for an overall response rate of 38.5% (72).

A promising new drug effective in metastatic cSCC is cosibelimab (CK-301).

Preliminary safety and efficacy data from the multicohort study indicated that 114 patients with multiple tumor types were enrolled and treated with cosibelimab. The ORR was 47.4% (95% CI: 36.0–59.1%) among those treated with cosibelimab.

Frequent AEs included fatigue (25%), anemia (21%), rash (18%), and nausea (16%) (73). Another promising drug combination currently in a phase II study is RP1 and nivolumab. RP1 is a genetically modified herpes simplex type 1 virus that is designed to directly destroy tumors and generate an anti-tumor immune response. Considering a total of 30 patients, 15 had CSCC, 4 had BCC, and 4 had MCC. Objective response was observed in 9 patients (60.0%) with CSCC, 1 (25%) with BCC, and 3 (75.0%) with MCC. Only patients with CSCC achieved a complete response, while none of the patients in the other subtype categories experienced a complete response (74). Currently, platinum-based therapy could be used for relapsed and/or metastatic spinocellular tumors for which there are no longer curative targets, according to AIOM guidelines 2021.^1^

Table 3 lists the main ongoing trials of BCC and experimental therapies.

**Table 3:** Ongoing trials in CSSC.

**Trial**	**Design**	**N' of patients**	**Indication**	**Intervention**	**ORR**
***NCT02883556 (CARSKIN)***	Phase II	57	Unresectable CSCC	Pembrolizumab 200 mg, administered as intravenous (IV) infusion every 3 weeks up to 24 months or until progression or unacceptable toxicity develops.	ORRW15 41%
***NCT02721732***	Phase II	202	Unresectable/mCSCC	Pembrolizumab IV over 30 min on day 1. Treatment repeats every 21 days for up to 24 months in the absence of disease progression or toxicity. Patients with clinical response or disease stabilization may continue treatment for up to an additional 12 months.	32%
***NCT02964559***	Phase II	11	laCSCC/mCSCC	Pembrolizumab IV over 30 min on day 1. Courses repeat every 3 weeks in the absence of disease progression or unacceptable toxicity.	
***NCT03284424*** ***(MK-3475-629/KEYNOTE-629)***	Phase II	159	laCSCC/mCSCC/recurrent CSCC	Pembrolizumab 200 mg via intravenous (IV) infusion on Day 1 of each 3-week cycle for up to ~2 years.	
***NCT03057613***	Phase II	18	Resected H&N CSCC	IMRT 60-66Gy for 6 weeks in combination with Pembrolizumab every 3 weeks for 16 weeks	
***NCT03833167*** ***(MK-3475)***	Phase III	570	Resected high-risk CSCC	Arm A: 400 mg pembrolizumab by intravenous (IV) infusion administered on Day 1 of each 42-day cycle (Q6W) for up to 9 cycles. Participants that complete 9 cycles of pembrolizumab and experience biopsy-proven-disease recurrence may be eligible to receive up to 18 additional cycles of pembrolizumab in an open-label design.	
				Arm B: placebo by IV infusion administered on Day 1 of each 42-day cycle (Q6W) for up to 9 cycles. Participants treated with placebo who experience biopsy-proven-disease recurrence may be eligible to receive up to 18 cycles of pembrolizumab in an open-label design.	
***NCT03969004***	Phase III	412	Resected high-risk CSCC	Arm A: Cemiplimab Intravenous (IV) infusion over 30 minutes	
				Arm B: Placebo Intravenous (IV) infusion over 30 min	
***NCT03834233*** ***(CA209-9JC)***	Phase II	24	laCSCC/mCSCC	Nivolumab 3 mg/kg IV every 14 days until disease progression, unacceptable toxicity or up to 12 months.	
***NCT04204837***	Phase II	31	laCSCC/mCSCC	Nivolumab will be given on Day 1 of every 14-day cycle (Q2W) at a dose of 240 mg as an IV infusion until progression, unacceptable toxicity or discontinuation for other reasons for up to 2 years.	
				Arm B: Nivolumab IV over 30 min and ipilimumab IV over 30 min at week 0. Treatments repeat every 2 weeks for nivolumab and 6 weeks for ipilimumab for up to 1 year in the absence of disease progression or unacceptable toxicity.	
***NCT03565783***	Phase I	40	H&N CSCC	Cemiplimab IV over 30 min every 3 weeks. Cycles repeat every 3 weeks for up to 6 weeks with or without radiation therapy at the discretion of the treating physician in the absence of disease progression or unacceptable toxicity.	
***NCT04807777***			Solid organ transplant recipients with advanced CSCC		
***NCT04349436 (ARTACUS)***			Liver/Kidney transplant recipient with advanced CSCC		
***NCT03684785***			Advanced MCC/advanced CSCC		
***NCT04799054***			Locally advanced/metastatic solid tumors		
***NCT04596033 (TiTAN-1)***			Advanced solid tumors including CSCC		
***NCT03458117 (20139157 T-VEC)***			Locally advanced NMSC		
***NCT02978625***			Advanced/refractory NMSC		
***NCT04160065***			Advanced CSCC/MCC		
***NCT04502888***			Advanced CSCC		

## MCC

Treatment of Merkel carcinoma depends on the disease's location and extent. The first treatment of choice is surgery, while the second treatment of choice is radiotherapy.

FDA-approved anti-PD-1/PD-L1 drugs are pembrolizumab and avelumab. PD-1/PD-L1 inhibitors achieve responses in 50% of patients with advanced disease in a durable and manageable safety profile (75).

The efficacy and safety of Avelumab as second-line treatment for mMCC have been studied in the single-arm, multicenter, phase II clinical trial JAVELIN Merkel 200: 88 patients treated with avelumab after not responding to chemotherapy had been followed up for a median of 40.8 months. The ORR was 33.0%, with complete response in 11.4% (10 patients), while the median duration of response was 40.5 months. Grade ≥3 treatment-related adverse events occurred in 11.4% of patients, and 21.6% of patients developed an immune-related adverse event (6).

The FDA approved pembrolizumab in the treatment of adult and pediatric patients with recurrent locally advanced or metastatic MCC, based on Cancer Immunotherapy Trials Network protocol 9 (CITN-09). In this multicenter phase II trial, 50 adults with no previous systemic therapy for advanced MCC received pembrolizumab for up to 2 years. The median follow-up time was 14.9 months. The ORR was 56% (complete response: 24% and partial response: 32%). The 24-month OS rate was 68.7%. Grade 3 or greater treatment-related adverse events occurred in 14 out of 50 patients (28%), leading to treatment discontinuation in 7 out of 50 patients (14%) (76).

A recent systematic review showed that the immunotherapy response to MCC is independent of the presence of Merkel cell polyomavirus (MCPyV).

The ORRs were 41.02% (32 of 78) and 41.66% (20 of 48) for MCPyV-positive and -negative tumors, respectively, with no difference in response (OR, 1.10; 95% CI, 0.52–2.33) (77).

A recent study has shown that there are also immunological differences between MCPyV-positive and MCPyV-negative MCCs.

T-cell clonality is much higher intratumorally, and there are far more neoantigens in MCPyV-positive tumors than in tumors without the virus, yet there is evidence of a response to immunotherapy for both tumor types. New therapeutic strategies involving immunotherapy in an adjuvant or neoadjuvant setting and MCPyV-specific adoptive T-cell transfer may lead to new information that can be used in clinical practice (78).

Table 4 lists the main ongoing trials of BCC and experimental therapies.

**Table 4:** Ongoing trials in MCC.

**Trial**	**Design**	**N' of patients**	**Indication**	**Intervention**
**NCT02488759** ***(CheckMate358)***	Phase I/II	578	Virus-associated diseases (MCC)	Nivolumab intravenous infusion as specified - Nivolumab intravenous infusion as specified - Nivolumab intravenous infusion as specified with Ipilimumab intravenous infusion as specified - Nivolumab intravenous infusion as specified with Relatlimab intravenous infusion as specified - Nivolumab intravenous infusion as specified with Daratumumab intravenous infusion as specified
***NCT03712605*** ***(STAMP)***	Phase III	280	Resected MCC	Arm A: Pembrolizumab IV over 30 min on day 1. Treatment repeats every 21 days for up to 17 cycles in the absence of disease progression or unacceptable toxicity. Patients may also undergo standard of care radiation therapy within 14 days of day 1, cycle 1.
				Arm B: standard of care observation every 3 months for 1 year, and then every 6 months for 5 years. Patients may also undergo standard of care radiation therapy within 14 days of day 1, cycle 1.
***NCT03271372*** ***(ADAMI)***	Phase III	100	Resected MCC with nodal metastasis	Arm A: Avelumab IV over 1 h once every 15 days for the first 120 days (Induction Phase 1), once every 30 days for the next 120 days (Induction Phase 2), and then once every 120 days (Maintenance Phase) for a maximum of 720 days (~24 months or 2 years total) in the absence of disease progression or unacceptable toxicity.
				Arm B: placebo IV over 1 h once every 15 days for the first 120 days (Induction Phase 1), once every 30 days for the next 120 days (Induction Phase 2), and then once every 120 days (Maintenance Phase) for a maximum of 720 days (~24 months or 2 years total) in the absence of disease progression or unacceptable toxicity.
***NCT04291885*** ***(I-MAT)***	Phase II	132	Resected MCC	Arm A: 6 months of Avelumab at a dose of 800 mg as a 60-min intravenous (IV) infusion once every 2 weeks (13 doses)
				Arm B: 6 months of Placebo as a 60-min intravenous (IV) infusion once every 2 weeks (13 doses)
***NCT02196961*** ***(ADMEC-O)***	Phase II	180	Resected MCC	After complete resection of Merkel cell carcinoma, patients randomized to the treatment arm will receive nivolumab at a fixed dose of 480 mg by IV infusion every 4 weeks for up to 1 year (i.e., 13 doses).
***NCT03798639***	Phase I	7	Resected MCC	Arm A: Nivolumab IV over 30 min at week 0. Treatments repeat every 4 weeks for 1 year in the absence of disease progression or unacceptable toxicity. Beginning week 2, patients also receive radiation therapy on Monday-Friday or 5 days per week for 6 weeks in the absence of disease progression or unacceptable toxicity.
				Arm B: Nivolumab IV over 30 min and ipilimumab IV over 30 min at week 0. Treatments repeat every 2 weeks for nivolumab and 6 weeks for ipilimumab for up to 1 year in the absence of disease progression or unacceptable toxicity.
***NCT03684785***			Advanced MCC/advanced CSCC	
***NCT04799054***			Locally advanced/metastatic solid tumors	
***NCT02465957 (QUILT-3.009)***			Advanced MCC	
***NCT03747484***			Unresectable/metastatic MCC	
***NCT03458117***			Locally advanced NMSC	
***NCT02819843***			Solid tumors with skin metastasis, including MCC	
***NCT02978625***			Advanced/refractory NMSC	
***NCT04160065***			Advanced CSCC/MCC	

## Basosquamous carcinoma

Basosquamous carcinoma (BSC) is a rare and aggressive NMSCs, being an intermediate entity between BCC and SCC.

According to the “WHO classification of skin tumors, BSCs are BCCs associated with squamous differentiation” (79). However, their ability to metastasize seems more like SCCs than BCCs (80–82).

There are currently no treatment guidelines for the treatment of BSCs. However, according to most authors, wide surgical excision is the first treatment of choice, with a recurrence rate of as much as 45% but reduced if Mohs surgery is used (83–85).

On the other hand, the cases of 4 patients with locally advanced BSC treated effectively with vismodegib have been reported recently. All lesions went into complete remission, which in two cases was maintained for a very long time (86–88).

In view of this data, there would seem to be a new treatment frontier for difficult-to-treat surgically treated BSCs, which should be evaluated in controlled trials (89).

Moreover, the cases of two patients with inoperable and non-treatable radiotherapy laBSCs, which were successfully treated with sonidegib 200 mg daily, have recently been published. In the first case, after 4 months of treatment, a reduction of more than 50% of the tumor mass was described in the absence of adverse events. In the latter, the same result was achieved after only 3 months, with complete remission at 6 months of therapy, at the cost of mild adverse events treated with brief, temporary discontinuation of the drug (90).

## New perspectives in NMSCs

### Intratumoral therapy in NMSCs

NMSCs enable a locoregional approach and treatment.

Treatments involving injections of drugs that can stimulate the immune system are emerging.

The goal is to develop a powerful immune response against the tumor while also acting at a distance through the abscopal effect. Two of the most promising molecules used in combination with immunotherapy are cavrotolimod (a Toll-like receptor 9 agonist) (91) and a Toll-like receptor 7/8 agonist (92). These molecules with high immunogenic potential have the role of inducing a potent antigenic response.

Other promising immunomodulatory drugs are Ifa-Hu 2.0 and SL-172154.

The former is an autologous vaccine with high immunogenicity given by bacterial antigens that can stimulate a potent cytotoxic response, and the latter is a fusion protein that simultaneously activates TNF and inhibits the CD47/Sirpa checkpoint (93). Ultimately, cancer viruses are also considered in the treatment of NMSC.

Talimogene laherparepvec (T-VEC) is an attenuated herpes simplex type 1 virus encoding granulocyte-macrophage colony-stimulating factor, which is designed to preferentially replicate in tumor cells, enhance antigen loading of MHC class I molecules, and induce antitumor immune responses. T-VEC is analyzed in several studies in combination with radiotherapy or immunotherapy (94).

### Cell-based immunotherapy in NMSCs

Immune cells associated with immune-stimulating drugs are another area of study. For example, Merkel carcinoma evades immunosurveillance by downregulating HLA class 1. A phase II study is evaluating the efficacy of natural killer cells stimulated by an IL-15 analog to increase the effectiveness of immune cells (95). Another approach of great interest is the use of personalized cell therapy that uses tumor neoantigens to stimulate CD4/CD8 cells to recognize MCC. CD4/CD8 cells express high-affinity receptors for MCPyV that combined with immunotherapy are studied in phase I/II trials. A response was obtained in 4 patients, and one of them achieved a complete response (96).

### Immunotherapy in the adjuvant setting

Adjuvant immunotherapy in stage III melanoma led to an improvement in recurrence-free survival (97). The good results obtained on melanocytic pathology have led to an increased interest in immunotherapy in adjuvant settings, also for high-risk NMSCs. Several studies have combined pembrolizumab and cemiplimab after surgery or radiotherapy or in combination with radiotherapy in NMSCs (NCT03969004, NCT03057613, and NCT03833167). Other phase II and III studies are evaluating PD-1 and anti-PD-L1 in MCCs after surgical excision (NCT03712605, NCT03798639, NCT03271372, NCT04291885, and NCT02196961).

### Best prospects in the neoadjuvant setting

In some cases, because of tumor extent or location, surgery or radiation therapy cannot be used as first-line treatments; therefore, the role of pharmacologic therapies in NMSCs before surgery is currently a field of study of considerable interest.

Currently, targeted therapy with sonic hedgehog inhibitors for BCC and immunotherapy for NMSCs (in particular, cSCC and MCC) seem to be the best prospects for neoadjuvant treatment. Unfortunately, none of these approaches with efficacy demonstrated by case reports, case series, or randomized trials have currently gained indication as neoadjuvant therapy for NMSCs (98).

More specifically, neoadjuvant treatment with cemiplimab in CSCCs and nivolumab in MCCs show promising results (NCT03565783, NCT04428671, and NCT02488759).

However, regarding targeted therapy in view of its efficacy in decreasing tumor burden, vismodegib could be very promising in the neoadjuvant phase.

In 15 patients with la-BCC treated with vismodegib in the neoadjuvant phase, after a follow-up of 22 months, the size of the final surgical defect has been reduced by 34.8% from baseline, and only 1 tumor has recurred (99).

Other studies investigating vismodegib in the same setting are ongoing (NCT03035188 and NCT02667574).

Sonidegib, followed by surgery or imiquimod, is also currently being evaluated in the neoadjuvant setting for la-BCC (NCT03534947).

## Conclusion

Immunotherapy and targeted therapy have revolutionized advanced skin cancer disease's survival and treatment of these diseases. Despite the successes of these new drugs, the new future challenges will be to select patients who will benefit from these treatments. Biomarkers and tumor microenvironment parameters will have increasing importance in clinical decision-making and more precise and personalized medicine. Clinical trials are critical to increasing knowledge of NMSCs and melanoma. More and more information is rapidly moving from the laboratory to the patient's bedside. But equally important will be real-life data from the clinical experience of centers treating advanced cutaneous neoplasms to consolidate data from registration studies and to evaluate the efficacy and toxicity of new molecules.

## Author contributions

Conceptualization: MR, NS, and SB. Methodology: NS and LM. Software: VP and LT. Validation and data curation: SR and PQ. All authors contributed to the article and approved the submitted version.

## Footnotes

^1^ https://www.aiom.it/linee-guida-aiom-2021-tumori-cutanei-non-melanoma
